# Dental opioid prescribing rates after the up-scheduling of codeine in Australia

**DOI:** 10.1038/s41598-020-65390-6

**Published:** 2020-05-21

**Authors:** L. Teoh, S. Hollingworth, R. Marino, M. J. McCullough

**Affiliations:** 1 Melbourne Dental School, University of Melbourne, Carlton, Victoria, Australia; 2 School of Pharmacy, University of Queensland, Woolloongabba, Queensland, Australia

**Keywords:** Dental public health, Oral analgesics

## Abstract

The misuse of pharmaceutical opioids is a major public health issue. In Australia, codeine was re-scheduled on 1 February 2018 to restrict access; it is now only available on prescription. The aim of this study was to measure the change in dental opioid prescriptions, one year before and after the codeine re-scheduling in Australia and to assess dental prescribing rates of opioids for 2018 by population and by clinician. Data was extracted for dental opioids for the year immediately prior and after the codeine up-schedule (1 February 2017-31 January 2019) from the publicly-available national prescription database (Pharmaceutical Benefits Scheme). Descriptive statistics, T-tests and odds ratios were used to identify significant prescribing differences. Codeine, codeine/paracetamol, oxycodone and tramadol use increased significantly the year after the codeine restriction than the previous year (13.8–101.1%). Australian dentists prescribed 8.6 prescriptions/1,000 population in 2018, with codeine/paracetamol accounting for most prescriptions (96%). The significant increase in opioid prescribing highlights that Australian dentists may be contributing to the misuse of pharmaceutical opioids. Educational efforts should be targeted at the appropriate use of opioids and patient selection. Dentists should be added to the prescription monitoring system SafeScript so they can make informed decisions for patients who are potentially misusing opioids.

## Introduction

Australia’s opioid crisis has been well documented; hospitalisations for pharmaceutical opioid poisoning surpassed those of heroin use and the misuse of pharmaceutical drugs as the most common reason for people to enter drug and alcohol treatment programs in Australia^1^. Pharmaceutical opioid deaths are now increasing at a greater rate than that due to heroin use as shown in a retrospective analysis from the National Coronial Information Systems between 2001 to 2012^2^, and three people die every day from drug-related events involving pharmaceutical opioids^3^. Forty percent of deaths due to prescription opioid use were associated with a valid prescription of oxycodone for non-cancer pain (2001 to 2011)^1^.

The main source of opioids for non-medical use is leftover tablets of legitimate prescriptions sourced through social networks such as family and friends^4^. In Australia, codeine products, both over the counter (OTC) and prescription, comprised the most commonly misused pharmaceutical products, followed by oxycodone and tramadol^5^. Codeine is the most frequently used opioid analgesic in Australia^6^, and ranks fourth behind methadone, oxycodone, and morphine in being implicated in pharmaceutical opioid overdose deaths^2^. Nearly three in five (56%) codeine-containing analgesic products were purchased OTC in 2013^7^. Prescription-only codeine utilisation was not accounted for in 21% of claims on Australia’s national medicines subsidy, the Pharmaceutical Benefits Scheme (PBS)^7^. A case series study reported that two in five (39%) patients with an opioid addiction were dependent on codeine alone^8^. The rate of codeine-related deaths increased several fold over ten years (3.5/million in 2000; 8.7/million in 2009)^9^. In response to the misuse, risks and toxicity, the Therapeutic Goods Administration (TGA) up-scheduled codeine from OTC and prescription to prescription only on 1 February 2018^10^.

Dentists in Australia have increasingly been prescribing opioids; the most common product is codeine with paracetamol (Panadeine Forte)^11^. This pattern continues despite non-steroidal anti-inflammatory drugs (NSAID) being more appropriate for dental pain^12,13^. NSAIDs, alone or in combination with paracetamol, were found to be the most effective for dental pain while minimising adverse effects in an overview of systematic reviews^14^. Irrespective, dental opioid prescribing increased 30% between 2013 and 2016^11^.

The legislative change to codeine scheduling appears to have reduced codeine misuse as highlighted in a recent analysis of monthly opioid exposure calls to the New South Wales Poisons Information Centre^15^. Additionally, studies of medical opioid prescribing indicate that there is no change in rates of dispensed opioid prescriptions after the legislative change compared to before (from January 2016 to December 2018)^16^. However, dental prescribing of opioids is increasing and some dentists are inappropriately preferencing the use of opioids over NSAIDs as recommended in the current Australian therapeutic guidelines^17^. The effect of the codeine rescheduling change on dental opioid prescribing is unknown. Thus, there were two aims of this study: 1) to assess prescribing rates of dental opioids in 2018 and 2) to analyse the dispensed use of dental opioids, one year before and one year after the up-scheduling of codeine.

## Method

This study involved an analysis of dispensed prescriptions to the Australian population from outpatient pharmacies from a nationally-representative database. The data used in this study was obtained from Medicare Australia administered by the Australian Government Department of Health. As the data was publicly available and aggregated, no ethical approval was required. The Strengthening the Reporting of Observational Studies in Epidemiology (STROBE) reporting guideline was followed.

### Data sources

The Department of Health provides a national database on prescribed medicines in Australia that are recorded under the Pharmaceutical Benefits Scheme (PBS)^18^. We analysed opioids subsidised by the Australian Government for dental prescribers listed on the PBS including codeine, codeine 30 mg/paracetamol 500 mg, hydrocodone, morphine, oxycodone, and tramadol. For the first part of the study, we assessed opioid use one year prior immediately prior to and one year after the change in codeine re-scheduling to prescription only (1 February 2018) by extracting monthly data of PBS dispensed use of dental opioids from 1 February 2017 to 31 January 2019. In Australia, the government subsidises the cost of medicines listed on the PBS. Some medicines (and for specific indications) are not subsidised and so the prescriber will issue a ‘private’ prescription where the consumer funds the full cost of the medicine. These are not recorded under PBS data and therefore are not accounted for in the analysis. PBS prescriptions account for approximately 75% of dispensed medicine use in Australia, where prescriptions are dispensed in hospital settings and outpatient pharmacies^19^. However, most dental prescriptions would be from community pharmacies, of which PBS subsidised prescriptions represent around 93% of community prescriptions^20^. Therefore, the majority of dental prescriptions (closer to 93%) would be accounted for in this analysis. The ‘date of supply’ dataset was accessed as this is recommended when analysing medicine use^19^. Given the variations in processing, the dataset was accessed six months after the end study date (i.e. 1 August 2019)^19^. Dentists are urged to follow the Australian therapeutic guidelines^17^ but they are free to prescribe whatever medicines they choose. Despite the PBS being a national formulary, the laws governing the supply and prescribing of medicines varies among the states of Australia. The relevant difference is that dentists in Queensland are prohibited from prescribing oxycodone^21^.

For the second part of the study, we assessed the dental prescribing rates by population and clinician from 1 January to 31 December 2018. The number of registered dental practitioners were obtained from the Australian Health Practitioner Regulation Agency^22^ and the population value from the Australian Bureau of Statistics^23^.

### Outcome measures

Three outcomes were measured: 1) dental opioid use for 2018 using the standardised defined daily dose (DDD)/1,000 inhabitants/day metric (see below); 2) prescribing rates for 2018 by population and by dentist; 3) the mean percentage difference for each individual opioid for the year before and after the rescheduling change.

### Statistical analysis

We extracted and analysed data using Microsoft Excel. Descriptive statistics were used to determine dental opioid use for 2018 by population and by clinician. Medicine use was calculated using the accepted and standardised metric of the DDD per 1,000 populations per day^18,24^. The DDD value for each medicine was obtained from the World Health Organization Collaborating Centre for Drug Statistics Methodology; it is the average maintenance dose per day for the main indication in adults^24^. The DDD metric is acceptable to reflect utilisation for the opioids listed except oxycodone, as the WHO DDD has been found to be two to seven times higher than actual doses used for oxycodone^25^. Therefore, for oxycodone only, doses were converted to oral morphine equivalents and the morphine DDD used to calculate the DDD/1000 population/day metric to indicate utilisation^26^. In Australia, the number of tablets per pack is standardised and most products are prescribed in PBS-specified packs.

T-tests (two-tailed) were used to identify significant differences between the number of opioid prescriptions one year before and after the re-scheduling change; p < 0.05 was considered significant. Odds ratios were also used to quantify the proportionate change for each opioid for the year after the codeine restriction compared to the previous year, relative to the increase the population. The mid-year population was used, with the data obtained from the ABS.

## Results

### Dental opioid dispensed use in Australia

There were 213,933 dispensed dental opioid prescriptions in 2018 in Australia. Australian dentists prescribed 8.6 opioid prescriptions/1,000 population, 12.1 prescriptions/dentist and 0.1 total DDD/1000 inhabitants/day. Australian dentists preferentially prescribed the codeine 30 mg/paracetamol 500 mg product (accounted for 205,189 prescriptions, 96% of opioid prescriptions), followed by oxycodone (6,050 prescriptions, 2.8% of opioid prescriptions) and tramadol (2,490 prescriptions, 1.2% of opioid prescriptions) (Table 1).

**Table 1:** Dispensed prescriptions for opioids in Australian in 2018 by population, number of dentists and defined daily dose per 1,000 population per day.

Opioid	Number of prescriptions	Percentage of prescriptions	Number of prescriptions/1000 population	Number of prescriptions/dentist	Total DDD/1000/DAY
Codeine	199	0.1	0.0	0.0	0.0
Codeine/Paracetamol	205,189	95.9	8.2	11.6	0.1
Hydromorphone	1	0.0	0.0	0.0	0.0
Morphine	4	0.0	0.0	0.0	0.0
Oxycodone	6,050	2.8	0.2	0.3	0.0
Tramadol	2,490	1.2	0.1	0.1	0.0
Total	213,933	100.0	8.6	12.1	0.1

Mid-year population for Australia 2018: 24,992,400.

### Trends in dispensed use of dental opioids

**Figure 1:**
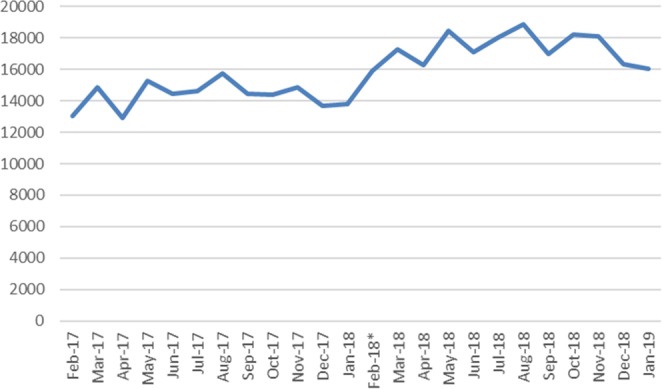
Dispensed dental prescriptions of codeine 30 mg/paracetamol 500 mg between 1 February 2017 and 31 January 2019. *Codeine was up-scheduled on 1 February 2018.

**Figure 2:**
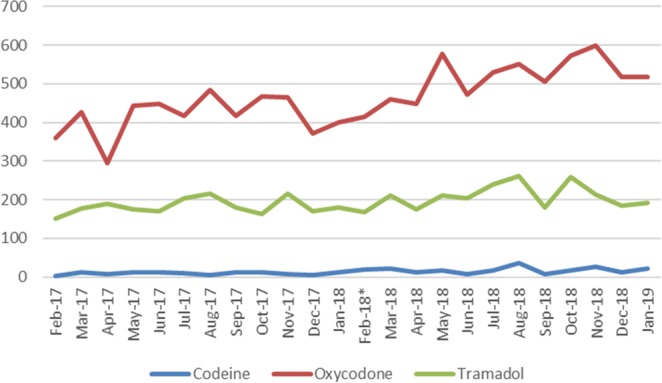
Dispensed dental prescriptions of codeine, oxycodone and tramadol between 1 February 2017 and 31 January 2019. *Codeine was up-scheduled on 1 February 2018.

When comparing the year after the up-scheduling of codeine to the previous year, there was a significant increase in the mean difference for the most commonly dispensed four opioids. The use of codeine, codeine/paracetamol, oxycodone and tramadol increased significantly over the two-year period (codeine: 101.1% mean increase from 8.7 to 17.5 prescriptions, p = 0.002; codeine 30 mg/paracetamol 500 mg: 20.6% mean increase from 14,337.0 to 17,284.5 prescriptions., p < 0.0001; oxycodone: 23.5% mean increase from 416.1 to 514.0 prescriptions, p < 0.001; tramadol: 13.8% mean increase from 183.1 to 208.3 prescriptions, p = 0.03). This occurred despite the increase in the population and number of clinicians over the same period being much lower (1.6% and 3.1% respectively). (Table 2, Figs. 1 and 2). The use of hydromorphone and morphine had low use. When comparing the year before and after the re-scheduling change, hydromorphone use stayed the same with one prescription dispensed both the year before and after the change; morphine use decreased from 15 prescriptions to 3 prescriptions respectively.

**Table 2:** Annual increase in dispensed opioids in Australia from 1 February 2017 to 31 January 2019.

Opioid	Mean No. prescriptions 1 Feb 2017-1 Jan 2019 (95% CI)	Mean No. prescriptions 1 Feb 2017-31 Jan 2019 (95% CI)	Annual % increase in mean	p-value	Odds ratio*
Codeine 30 mg	8.7 (6.7-10.6)	17.5 (12.5-22.5)	101.1	0.002	2.0
Codeine 30 mg with paracetamol 500 mg	14,337.0 (13,798.5-14,875.5)	17284.5 (16,639.6-17,929.4)	20.6	<0.0001	1.2
Oxycodone	416.1 (381.8-450.4)	514.0 (477.9-550.1)	23.5	<0.001	1.2
Tramadol	183.1 (170.4-195.8)	208.3 (188.5-228.2)	13.8	0.03	1.1

*The proportionate change of each opioid compared the total year after the re-scheduling change to the previous year.

When assessing the relative proportions of dispensed opioids, there was an increase in the relative proportions of opioids prescribed the year after the codeine restriction compared to the previous year, adjusted for the population increase. (codeine: OR: 2.0; codeine/paracetamol: OR: 1.2; oxycodone OR: 1.2; tramadol OR: 1.1).

## Discussion

This is the first study to assess the effect of the codeine up-schedule on dental opioid prescribing in Australia. There was a substantial increase in dispensed dental opioid use after the up-scheduling of codeine with relatively smaller increases in the population and clinician numbers. As expected, this was significant for codeine and codeine with paracetamol, but also evident for oxycodone and tramadol. This increase occurred despite the Australian therapeutic guidelines indicating that opioids are not to be used first line to treat dental pain^17^, and tramadol is also not recommended^17^.

Multiple studies have confirmed that NSAIDs alone or in combination with paracetamol are more effective than opioid combinations for dental pain^27^ and as such opioids are not first line treatment in the current Australian guidelines^17^. Moreover, analgesic doses of codeine provide no extra pain relief in the surgical extraction of third molars compared to paracetamol and ibuprofen alone^28^. Furthermore, patients undergoing third molar extractions who received the opioid combination products had significantly more adverse effects compared to the patients who received the NSAID combination products^27^. Given the limited role of opioids in dental pain and that the vast majority of dental conditions require active treatment alone where medicines would only be adjunctive^17^, theoretically, the dental prescribing of opioids should not be increasing at a rate greater than the population change. Previous longitudinal studies of dental opioid prescribing in Australia showed increases in the use of opioids^11^ but this does not explain the significant increase after the codeine up-schedule. About one in four dentists in Australia (16-27%) would preference the use of analgesics only (paracetamol, paracetamol/codeine, tramadol or oxycodone) for dental pain, instead of NSAIDs^29^. Australian dentists tend to recommend tramadol if their patient is allergic or finds codeine ineffective^30^, despite both medicines requiring transformation by cytochrome P450 2D6 to the active metabolites. The pharmacogenomic variability of cytochrome P450 2D6 is well established and therefore patients who have inherited two non-functional alleles of this enzyme will likely find both medicines ineffective. In addition, other non-clinical factors have been identified for prescribing by Australian dentists, such as patient pressure, fear of litigation, and the desire by dentists to make patients feel well managed^30^.

While the increase in the use of paracetamol/codeine may be partly accounted for by the up-scheduling of codeine, the large increase suggests that dentists may also be inappropriately prescribing opioids, or some people may be acquiring opioids from dentists for misuse^31^. A case series analysis of people who sought help for opioid dependence showed that many initiated codeine for the treatment of headaches and dental pain^8^. A previous study in Boston, USA showed that one in eight (12%) people who presented to the emergency department with backache, dental pain or headache were seeking to acquire an opioid drug^31^. A pre-filled opioid prescription, given prior to the extraction of wisdom teeth, is an independent risk factor for persistent opioid use^32^. Dental opioid prescriptions may be associated with subsequent opioid abuse in adolescents and young adults^33^.

Dental prescribing of opioids varies substantially across countries. Underscoring the findings from this analysis of dental opioid prescribing rates, a recent study by Suda *et al*. compared opioid prescribing between US and English dentists in 2016^34^. Australian dentists prescribe around four times less than those in the US (35.4 opioid prescriptions/1,000 US population versus 8.6 opioid prescriptions/1,000 Australian population) and almost five times less than those in the US when adjusting by clinician (58.2 opioid prescriptions/US dentist versus 12.1 opioid prescriptions/Australian dentist)^34^. In addition, US dentists prescribe a larger range of opioids and long-acting opioids than Australian dentists^34^. Notwithstanding this substantial difference to US dentists, Australian dentists prescribe 21 times more opioids than English dentists when adjusted for population (0.5 prescriptions/1,000 English population), and about ten times the rate when adjusting by clinician^34^. This vast difference in prescribing rates occurs despite all three countries displaying similar levels of oral health as measured by such as decayed, missing and filled teeth indexes, and levels of edentulousness^35^. Suda *et al*. noted that one major difference among prescribing practises is that English dentists are restricted to prescribe from a medicine formulary; they can only prescribe dihydrocodeine on the NHS^34^. Nevertheless, such a striking contrast must be questioned whether this is due to prescribing practices alone, or potentially opioid seeking behaviour across these different countries. Further, it may well be that there are much higher levels of opioid use in England as these medications are readily available over the counter and thus their use merely not measurable.

The limitations of this study are that the Australian PBS data only captures prescribing on the PBS and does not include prescriptions that are privately funded. Furthermore, no prescription would be generated where the dentist both prescribed and supplied the medicine directly to patients. It is expected that these sources of prescriptions will be relatively low; dental dispensing is rare in Australia. Prescription details such as dose, frequency and duration of treatment were not available therefore it was not possible to assess the appropriateness of the prescriptions. These data are not linked to morbidity or mortality data so it is not possible to ascertain any link between dental opioid prescribing and opioid misuse. Nevertheless, these data allowed for international comparisons and demonstrated prescribing trends in most of the population.

This study has substantial public health implications. It should initiate a call to action for dentists regarding their role in responsible opioid prescribing, as well as to re-evaluate the recommendations of opioids in dental practice. Given the established misuse of pharmaceutical opioids in Australia^5^ and other countries, and the associated public health burden, dentists should only prescribe opioids if NSAIDs and paracetamol have not been effective or cannot be tolerated; they should ensure a true therapeutic need exists. Continuing education efforts could be directed towards the appropriate use and limitations of opioids in general dental practice, with future research aimed at developing strategies to improve opioid prescribing among dentists at an individual level.

Strategies could be implemented for the early identification of people who are at higher risk of developing opioid dependence. Opioid-dependent individuals tend to also have co-morbidities of pre-existing chronic pain and psychiatric conditions^8^. High rates of concomitant prior mental health, substance use and chronic pain issues were recorded for these deaths due to codeine toxicity in a prospective study from the National Coronial Information System^9^. Given that the predominant source of drugs for misuse is mostly through leftover pills from legitimate prescriptions sourced through social networks^4^, manufacturers could consider the formulation of opioids in smaller pack sizes to reduce the number of leftover pills from a prescription.

One other measure to assist with the prescribing of medicines prone to misuse is the Safe Script program^36^, which is  obligatory in the Australian states of Victoria and Tasmania from April 2020. This initiative allows prescribers to access real-time prescription monitoring for all patients who acquire medicines prone to misuse from each prescriber and pharmacist to assist with ‘doctor shopping’ and to prevent the increased acquisition of these drugs from multiple clinicians. The initiative is only implemented in two Australian states and includes medical doctors, nurses, and pharmacists only. Given the significant increase in the use of codeine since the scheduling change, including dentists in the program will assist them to make more informed decisions when prescribing drugs of dependence to people who are seeking opioids for non-medical use.

## Conclusion

Underscoring the opioid crisis in Australia, this study illustrates how the increased prescribing of opioids by dentists, especially since the codeine restriction, may be contributing to the epidemic and how dental prescriptions can be a source of misuse. While guidelines are in place, further restrictions from the PBS such as prescribing authority prescriptions for selected indications and restricted quantities are some ways to curtail prescribing and limit the number of tablets prescribed. The prescription monitoring system - Safescript - should include dentists to allow them to be aware when prescribing opioids for people who acquire them for non-medical use. Consideration should be given when prescribing opioids for patients who have chronic pain, and procedures established for the early identification of people who may be prone to opioid dependence. Further research is needed to investigate the appropriateness of prescribing practices, and both undergraduate and continuing education of dentists should be targeted about the role of opioids in dental pain management.
